# Generation, maintenance and tissue distribution of T cell responses to human cytomegalovirus in lytic and latent infection

**DOI:** 10.1007/s00430-019-00598-6

**Published:** 2019-03-20

**Authors:** Sarah E. Jackson, George X. Sedikides, Georgina Okecha, Mark R. Wills

**Affiliations:** Division of Infectious Diseases, Department of Medicine, University of Cambridge, Addenbrookes Hospital, Level 5, Hills Road, Box 157, Cambridge, CB2 0QQ UK

**Keywords:** Human cytomegalovirus (HCMV), T cell memory, Inflation, Latency

## Abstract

Understanding how the T cell memory response directed towards human cytomegalovirus (HCMV) develops and changes over time while the virus persists is important. Whilst HCMV primary infection and periodic reactivation is well controlled by T cell responses in healthy people, when the immune system is compromised such as post-transplantation, during pregnancy, or underdeveloped such as in new-born infants and children, CMV disease can be a significant problem. In older people, HCMV infection is associated with increased risk of mortality and despite overt disease rarely being seen there are increases in HCMV-DNA in urine of older people suggesting that there is a change in the efficacy of the T cell response following lifelong infection. Therefore, understanding whether phenomenon such as “memory inflation” of the immune response is occurring in humans and if this is detrimental to the overall health of individuals would enable the development of appropriate treatment strategies for the future. In this review, we present the evidence available from human studies regarding the development and maintenance of memory CD8 + and CD4 + T cell responses to HCMV. We conclude that there is only limited evidence supportive of “memory inflation” occurring in humans and that future studies need to investigate immune cells from a broad range of human tissue sites to fully understand the nature of HCMV T cell memory responses to lytic and latent infection.

## Introduction

Primary infection with human cytomegalovirus (HCMV) in healthy individuals does not generally cause overt disease [1, 2]; however, a robust immune response is generated including neutralising antibodies and cellular responses which eventually controls and eliminates the lytic virus [3]. In the face of this immune response, the virus is not cleared probably due to the numerous viral immune evasion proteins encoded by the virus [4, 5] and is able to establish a latent infection in certain cell types [6]. Periodically the virus reactivates, resulting in antigenic stimulation of HCMV-specific secondary immune responses and generating distinct memory CD4 + and CD8 + T cell populations, characteristic of this infection (recently reviewed in [7]). The impact of HCMV in changing numerous immune parameters has been shown conclusively in a twin study, where identical twins varied in their HCMV infection status. It was shown that the HCMV seropositive twins had increased T cell effector memory populations and alterations in serum proteins [8].

Understanding how HCMV manipulates the immune response over time during both latent carriage and periodic reactivation of the virus leading to lytic infection requires an appreciation of the virus lifecycle. It has been shown that bone marrow resident CD34 + progenitor cells and CD14 + monocytes derived from these progenitors are sites of HCMV latent viral carriage in vivo [9]. Recent transcriptomic and single-cell studies have shown that latent infection is more dynamic than previously thought with a number of different transcriptional profiles of HCMV gene expression [10, 11];however, HCMV latent infection of CD34 + and CD14 + cells can still be characterised by the lack of infectious virion production. Previous studies have identified particular viral genes which are transcribed during latency and are functionally important for maintaining the latent infection, including UL138 [12, 13], LUNA (latent undefined nuclear antigen; UL81-82as) [14–16], US28 [17, 18], UL111A (vIL-10) [19, 20]. CD34 + cells latently infected in vitro with HCMV have an altered secretome which includes increased expression of chemokines that can attract CD4 + T cells as well as immune-suppressive cytokines IL-10 and TGF-β [21]. In addition, it has also been shown that CD4 + T cells specific to these HCMV proteins expressed during latency can secrete IL-10 as well as having anti-viral effector functions [22, 23]. Taken together this suggests that latent HCMV infection manipulates the immune response towards a more suppressive phenotype, which is in contrast to the predominantly anti-viral effector phenotype of CD4 + T cells specific to HCMV proteins expressed during lytic infection such as pp65,IE and gB [24].

It is important, therefore, to consider the impact of long-term carriage of HCMV, in some cases for many decades, on the immune response of the healthy host.

## Does memory inflation of CMV-specific T cell responses occur in humans?

Memory inflation is a phenomenon associated with cytomegalovirus infection; it has been extensively studied in the murine model of cytomegalovirus (MCMV) infection. The expansion of IE1-specific CD8 + T cells in MCMV infection was originally described in the lungs of latently infected mice [25]. This work also demonstrated that T cells specific for other MCMV proteins were non-inflationary (m04, M83 and M84). In addition the inflationary CD8 T cells had an effector memory phenotype and retained the ability to make IFN-γ upon restimulation. Subsequently another group described a similar observation using the term “memory inflation”, and this was observed in multiple organs and appeared to be driven by continuous activation of the T cells [26, 27]. The process of “memory inflation” has subsequently been observed by other laboratories in additional mouse strains, and a number of MCMV proteins have been identified as driving “inflationary” responses, including m38, m139 and IE3 [28–31], m164 [32, 33] and IE1/pp89 [25, 26, 33–35]. The term of “memory inflation” was not precisely defined in the original work using this term [26] and recently Paul Klenerman has published an updated definition with three parts: ‘(1) Restricted contraction following priming, leading to long-term maintained memory pool. (2) A dominant and sustained “effector-memory” phenotype. (3) A sustained effector functionality without features of immune exhaustion’ [36].

A characteristic of cytomegalovirus infection in both mice and men is the expansion of high-avidity T cell clonotypes specific to the virus; in mice these populations are preferentially selected to “inflate” [37]. The contraction of the T cell receptor (TCR) repertoire to focus on specific immunodominant HCMV protein epitopes has been observed in numerous studies over the last 2 decades [38], and the presence of these high-affinity clones in HCMV responses, directed towards particular epitopes from the tegument protein pp65 [39–41], has been used as evidence that memory inflation occurs in the human host as well as the mouse [37, 42]. The focusing of HCMV-specific CD8 + T cells on particular TCR sequences, generating high-affinity and -avidity clones is seen against the IE1 protein as well as pp65 [43, 44]. However, none of these studies show accumulation of these high-affinity clones within the CD8 + T cell compartment over time. Where longitudinal studies have been undertaken, the frequency of these clones has been shown to be relatively stable over a number of years [44], once the primary response has resolved (based on absolute numbers of CD8 + T cell-specific clones) [41]. Inflation of the frequency of HCMV-specific T cells has been implied from a number of cross-sectional studies where increasing age of the subjects has been taken as a surrogate marker of time of HCMV carriage [45–49]. These cross-sectional studies also demonstrated that many of these T cells remained polyfunctional despite having a highly differentiated phenotype [39, 44, 46, 50–54]. In addition it has been shown that the total CD8 + T cell pool (based on absolute numbers) expands to accommodate these CMV-specific expansions [52, 55]. Other studies have demonstrated a decline in the absolute numbers of naïve T cells in older age irrespective of CMV sero-status [23, 56]. These changes in absolute numbers of the T cell compartment may account for the observations of increased frequency of memory T cell populations in older CMV sero-positive donors [45–49] based on frequency of responses only.

The term inflation has been used in many human studies of CMV-specific T cell responses to refer to large percentages (generally greater than 1.5%) of tetramer or cytokine-positive T cells responding to particular HCMV peptide epitopes [57]. Certainly, there are a number of studies showing that in some donor cohorts, there are large proportions of memory T cell responses focussed on CMV in older donors, who have likely been infected for many years compared to younger donors [45–49, 58–60]. A limited number of studies have related this increase in magnitude of CMV-specific memory T cell responses to an increase in the absolute numbers of antigen-specific T cells present in donors [54, 61, 62]. The evidence from these studies has been used by some to support the hypothesis that an increase in the size of the CMV memory T cell population over time in humans is indicative of memory inflation. However, other studies have shown that large expansions in the frequency of CMV-specific memory T cells is a feature of being CMV positive irrespective of age, including studies of children as well as younger adults [51, 63–67], this is also true when observing the absolute size of the memory T cell compartment [23, 52].

**Fig. 1:**
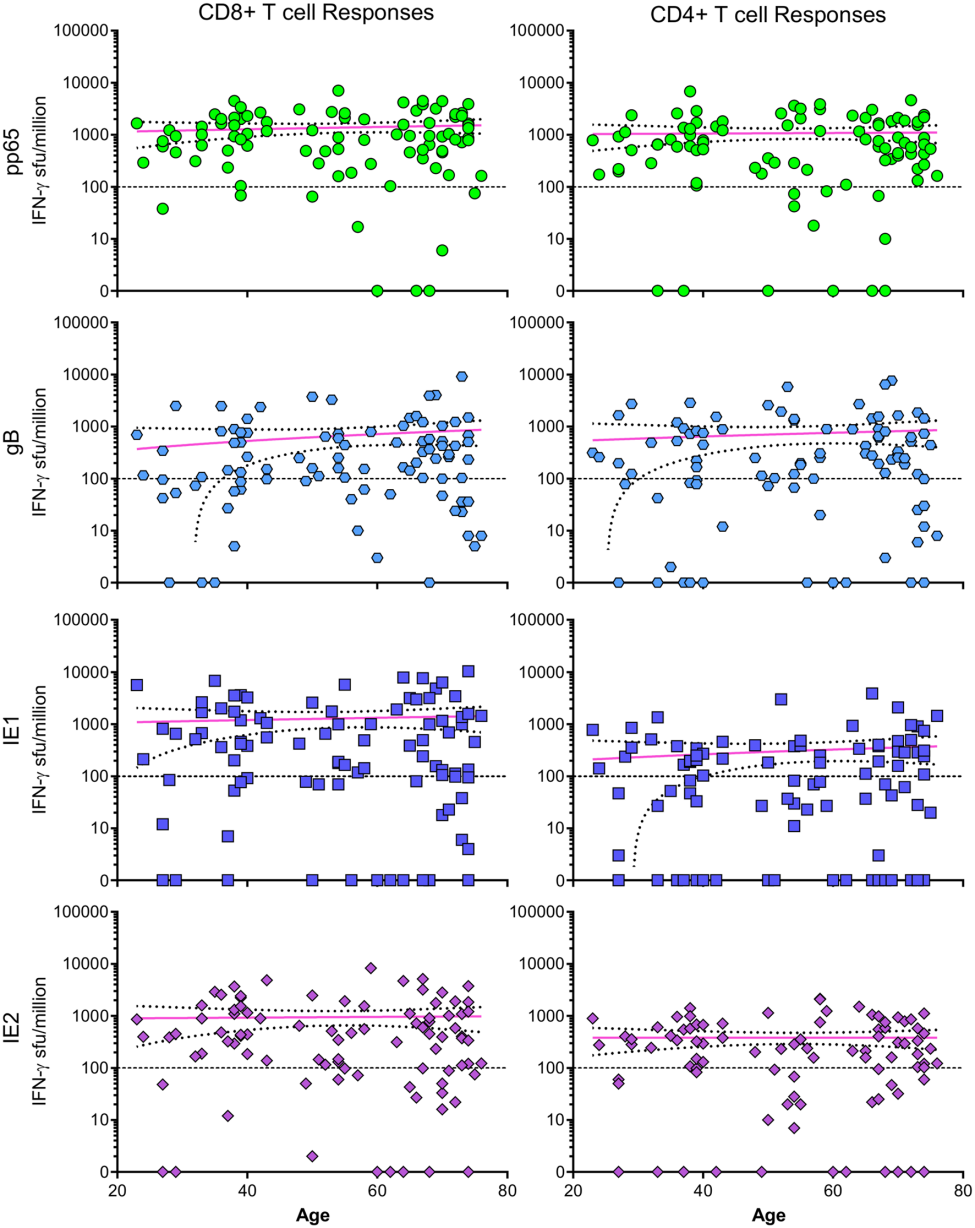
CD4 + and CD8 + T cell IFN-γ responses to pp65, gB, IE1 and IE2 HCMV proteins. The T cell IFN-γ responses measured by FluoroSpot versus donor age from the ARIA study [23] to four lytic expressed HCMV proteins pp65 (green), gB (light blue), IE1 (dark blue) and IE2 (purple) are shown. There is no significant correlation (Spearman rank correlation test) between the magnitude of the IFN-γ response with donor age for any of the four proteins shown

Previously, we wished to investigate whether T cell responses to HCMV proteins expanded as a result of long-term carriage of the virus [23]. To address this question, we have also used a cross-sectional age donor cohort to investigate whether memory CD4 + and CD8 + T cell responses expand as a result of long-term viral carriage. We used pools of peptides spanning six HCMV proteins that are expressed in lytic infection (lytic antigens) that have been shown to stimulate responses in previous studies [24, 68–70]. We also included pools of peptides spanning HCMV proteins UL138, LUNA, vIL-10 and US28, which have been shown to be expressed by latently infected cells (latent antigens). In the paper, we presented the cumulative IFN-γ responses to these lytic and latent antigens, the results showed that there was not an increase in either the breadth or magnitude of these T cell responses with increasing age. Looking at the frequency of T cell responses to four immunodominant HCMV proteins (pp65, gB, IE1 and IE2) in relation to the age of the donors, clearly shows that both young and old donors can have large expansions of both CD4 + and CD8 + T cell memory responses to these lytic proteins (Fig. 1).

The main drawback with using cross-sectional age studies to try and identify inflationary memory responses in human subjects is the many unknowns in disease aetiology of the donors compared to murine studies. For instance, in humans, the time of primary infection and the initial infectious dose are not known. Also there is wide genetic variability in human subjects compared to inbred mouse strains such as C57BL/6 or BALB/c, commonly used in MCMV memory inflation studies [26, 28, 30, 32, 34, 37, 71–73].

Longitudinal studies of individual donors where multiple samples over time have been examined would clearly be a better approach for observing whether HCMV-specific memory inflation occurs in humans, albeit technically challenging. There have been a limited number of studies where longitudinal samples have been included; the results have provided evidence both for and against memory T cell inflation. In an earlier study, we investigated CD8 + T cell IFN-γ responses to HCMV in a small donor cohort where the frequency of T cells specific to ten different HCMV proteins was measured in the same individuals over a period of 3 years [68]. In this study, we did not observe inflation of the CD8 + T cell functional responses, indeed despite fluctuation in the magnitude of the response at the different time points, the responses were mostly stable. Fluctuations in the magnitude of HCMV responses have also been observed in another study over a period of 6 months; however, this is a short period of time and there was no net increase in T cell frequency [74]; fluctuations in the magnitude of memory responses over time are also observed in murine models [32]. In an Italian cohort of 25 older donors, the magnitude of pp65 and IE1 specific IFN-γ T cell responses (based on absolute counts of peripheral blood) was measured over a 5 year period. There was a significant increase in the magnitude of the CD8 + T cell pp65 response, but the IE-specific response remained mostly stable. There were no significant changes in the CD4 + T cell pp65 and IE1 responses although there was an increase in the mean responses at the later time point [75]. Investigation of a CD4 + T cell HLA-DR7 restricted gB epitope in two HIV-positive donors over 12 years, revealed stable responses to this epitope over time. However, the frequency of the epitope-specific CD4 + T cells at the start was 16% and 18%, and as such already a highly expanded memory CD4 + T cell population [57]. Investigation of the prevalence of a γδ T cell subtype, Vδ2^neg^, in CMV infection showed an “inflation” in this population in a cross-sectional age study but a longitudinal study measuring the absolute size of the Vδ2^neg^ population of certain donors did not show any increases over time [76]. A recent study investigating CD8 + T cell HLA-C restricted responses does give evidence supportive of memory inflation, HLA-Cw0702 epitopes from the UL28 and IE1 proteins dominated the CD8 + T cell pool in older donors (averaging 37% of total CD8 + T cells), and in longitudinal samples from some donors there does appear to be an increase in the frequency of this response to immediate early expressed proteins [77].

Within healthy adult populations, from whom many of these studies draw their participants, the unknown factor is when an individual was infected by HCMV. It is possible that the period when memory inflation occurs post-infection may be missed, as within the mouse model, inflationary responses are seen within 1 year of infection [26]. Therefore, it is worth investigating whether memory inflation is observable in primary HCMV infection. Due to the generally asymptomatic nature of HCMV infection in the majority of healthy individuals, finding and following primary natural HCMV infection is not straightforward. One approach has been the use of a HCMV sero-positive donor kidney being transplanted into a HCMV sero-negative recipient and the subsequent viral infection, replication and primary immune responses are then followed in the recipient. Using this model, it has been possible to elucidate the dynamics of both CD4 + and CD8 + T cell responses to HCMV and many of these patients have been followed for up to 5 years post-transplant, as such this covers a similar time period as the murine studies of memory inflation. Key findings from this work include the importance of CD4 + T cell responses in preventing symptomatic disease, and that effector CD8 + T cells arise later but can be directly cytotoxic towards CMV presenting cells [78]. This model has also been used to track the differentiation phenotype of CMV-specific T cells, including the loss of co-stimulatory molecules CD27 and CD28 [79]. Many of the published studies using this model have tracked the magnitude of CMV-specific T cells or tracked specific TCR clones (using both absolute counts and frequency measures), the results from these studies have provided no direct evidence of memory inflation of the CMV-specific T cells [78, 80–83]; however, one should remember that these patients were undergoing varying degrees of immunosuppression. In a limited number of studies where community-acquired primary HCMV infection has been identified in normally healthy individuals, it has been shown that CMV-specific CD8 + T cells acquire an effector phenotype during the acute phase of the infection and the numbers of CD8 + T cells increase (likely due to lymphocytosis, characteristic of natural CMV infection [1]); however, by 9 months post-infection these effects are mostly resolved, although an effector memory phenotype persists [84]. In earlier studies looking at the clonality of the CMV-specific CD8 + T cell response in primary infection, pp65-specific responses mostly remain stable [41, 59], but the frequency of IE1-specific responses may increase up to 104 weeks post-infection [59].

Due to the difficulties in studying the development of memory T cell responses to cytomegalovirus in humans, the use of surrogate markers, often translated from the mouse model to identify “inflationary” populations has been used. The fractalkine receptor, CX3CR1, has been used to classify different memory CD8 + T cell populations in both mouse and human. Expression of CX3CR1 by CD8 + T cells identifies a memory population with cytotoxic effector functions independent of other differentiation markers associated with tissue homing and other previously defined memory populations [85]. In MCMV infections, CX3CR1 has been shown to be a marker expressed by intermediate peripheral memory CD8 + T cells, which are generally “inflationary” populations. A similar population of memory CD8 + T cells expressing CX3CR1 has been identified in HCMV-infected humans and this population was observed in response to an adenovirus-based vaccine trial, suggesting this chemokine receptor could be used to identify “memory inflation” populations in humans [86]. CD85j (LIR1) expression on CD8 + T cells has been postulated to identify a memory inflation population in HCMV infection [87]. The authors demonstrated that there was an increase in the total CD8 + population expressing CD85j in older donors, and that CMV sero-positivity led to a 19% increase in expression overall. In murine studies, inflationary memory CD8 + T cell populations have an Effector Memory (EM) phenotype (defined as CD44^hi^, CD62L −, KLRG1 +), in humans a similar EM population (defined as CCR7 −, CD28 −, CD27 −, CD45RA −) can be identified within CMV tetramer-specific CD8 + T cells [29]. Increased numbers of CMV-specific EM cells results in increased numbers of CMV-specific CD8 + T cells overall, this was also demonstrated in the primary kidney transplant model where the proportion of EM Tetramer+ (pp65)-specific CD8 + T cells accumulates over time post-transplant [29].

**Fig. 2:**
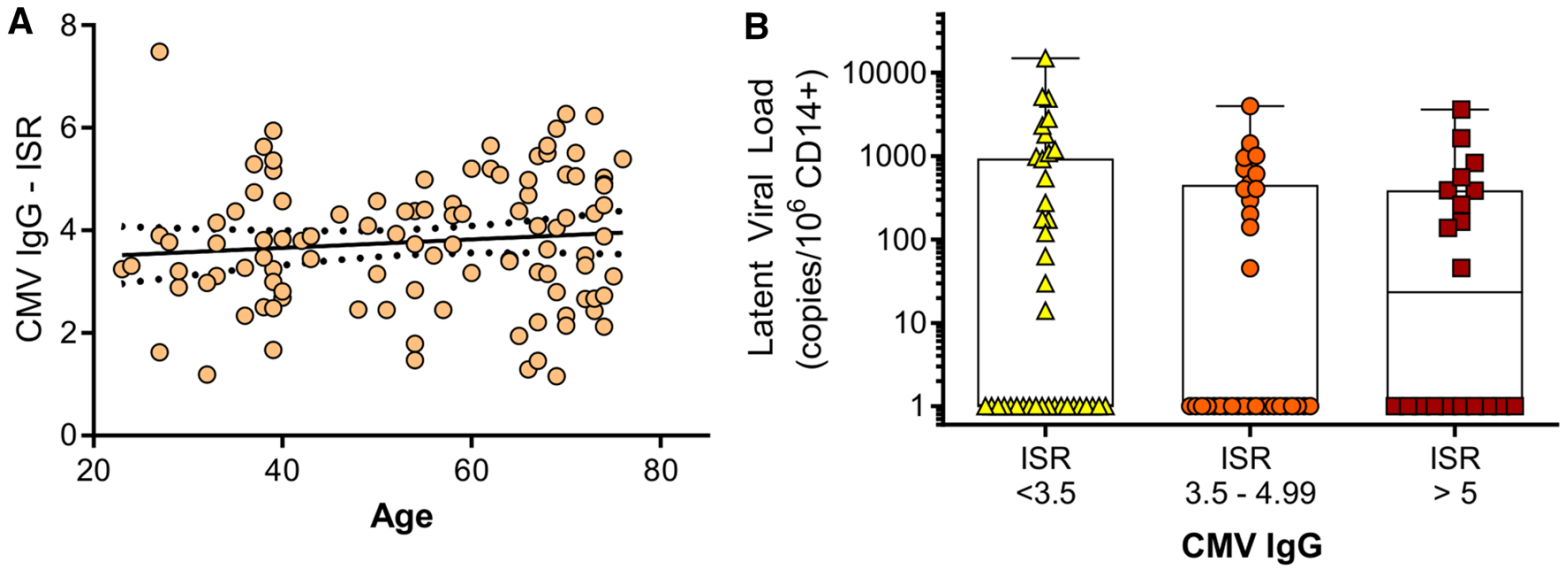
CMV IgG response related to age and latent CMV load in CD14 + monocytes. Serum HCMV IgG levels [immune system ratio (ISR)] from the ARIA study [23] related to donor age (**a**); there is no significant correlation (Spearman rank correlation test). Latent CMV load in CD14 + monocytes measured by droplet digital PCR as previously described [23] is shown related to low (< 3.5 ISR), medium (3.5–4.99 ISR) and high (> 5 ISR) CMV IgG levels (**b**); there is no significant difference in the magnitude of the latent viral load between the three groups (Kruskal–Wallis ANOVA test and Dunn’s multiple comparison post-test)

In addition to measuring T cell responses to CMV in cross-sectional ageing studies, the HCMV-specific humoral response has often been assessed. There is an increase in B cell numbers in CMV-positive older donors [88], which could result in increased secretion of antigen-specific immunoglobulins. A number of studies have shown that CMV-specific IgM [89] and IgG titres [89–92] were significantly increased in older donors. In a longitudinal study of older donors CMV IgG titres increased significantly over 5 years [75] and in donors aged over 85 years and in poor health increased IgG significantly correlated with loss of cognitive function [61]. It has been previously suggested that high CMV IgG levels correlate with high CMV-DNA levels [93]; therefore, we analysed whether there was a relationship between IgG titres and CMV latent viral carriage in CD14 + monocytes using the data from the donor cohort published in 2017 [23]. Within our donor cohort we did not observe an increase in latent viral load with donor age [23], in contrast to a previous study which did see an increase in latent viral load in donors over 70 years old [90]. Neither did we see an increase in CMV IgG in relation to donor age (Fig. 2a), in contrast to previous studies of CMV IgG titres. We further analysed the data set to see if CMV IgG levels were related to CMV latent viral load by banding the CMV IgG measure into (1) low responses, (2) medium responses and (3) high responses; as shown (Fig. 2b), the magnitude of the CMV IgG response did not relate to latent viral load carriage.

Overall, it is our view that there is not sufficient evidence to support the assertion that cytomegalovirus-specific “memory inflation” occurs in humans, in contrast to the work in the murine field. This is due to the problems in conducting such long-term longitudinal studies in humans combined with the difficulty of knowing when primary HCMV infection occurred. It is clear that younger people can have large HCMV-specific T cell expansions and it might be that the main part of the inflationary stage has already occurred at the point that these individuals have been first studied. It is interesting to note that the strongest evidence for “memory inflation” in humans has come from longitudinal studies measuring CD8 + T cell responses to immediate early HCMV proteins as first noted in the murine studies [59, 77].

## Is there evidence of memory inflation of CD4 + T cells secreting IL-10 in response to HCMV?

Within the mouse model of “memory inflation” the focus has mainly been on CD8 + T cell responses, less work has focussed on the CD4 + T cell compartment. There have been limited reports of memory CD4 + T cell inflation in MCMV, with evidence that CD4 + T cell responses directed to the m09 protein arise at later time points post-infection and are slightly inflationary [94]. In the murine model, CD4 + T cells are important in controlling persistent MCMV infection in the salivary gland, by killing infected cells [95]. The salivary gland environment in MCMV infection has also been shown to contain IL-10 secreted by CD4 + T cells which prevents MCMV clearance [96, 97]. The generation of a suppressive environment by CMV may be one method that the virus uses to modulate its environment to persist in myeloid lineage cells [4], certainly we have shown that latent infection of CD34 + cells results in the production of cellular IL-10 alongside the HCMV homolog vIL-10 (UL111a) and that these secretomes inhibit T cell effector function [21]. HCMV-specific cells that secrete IL-10 or have a regulatory phenotype have been identified by ourselves and other research groups [22, 98–100]; however, whether these T cells are inflated in CMV infection has not been definitively investigated.

**Fig. 3:**
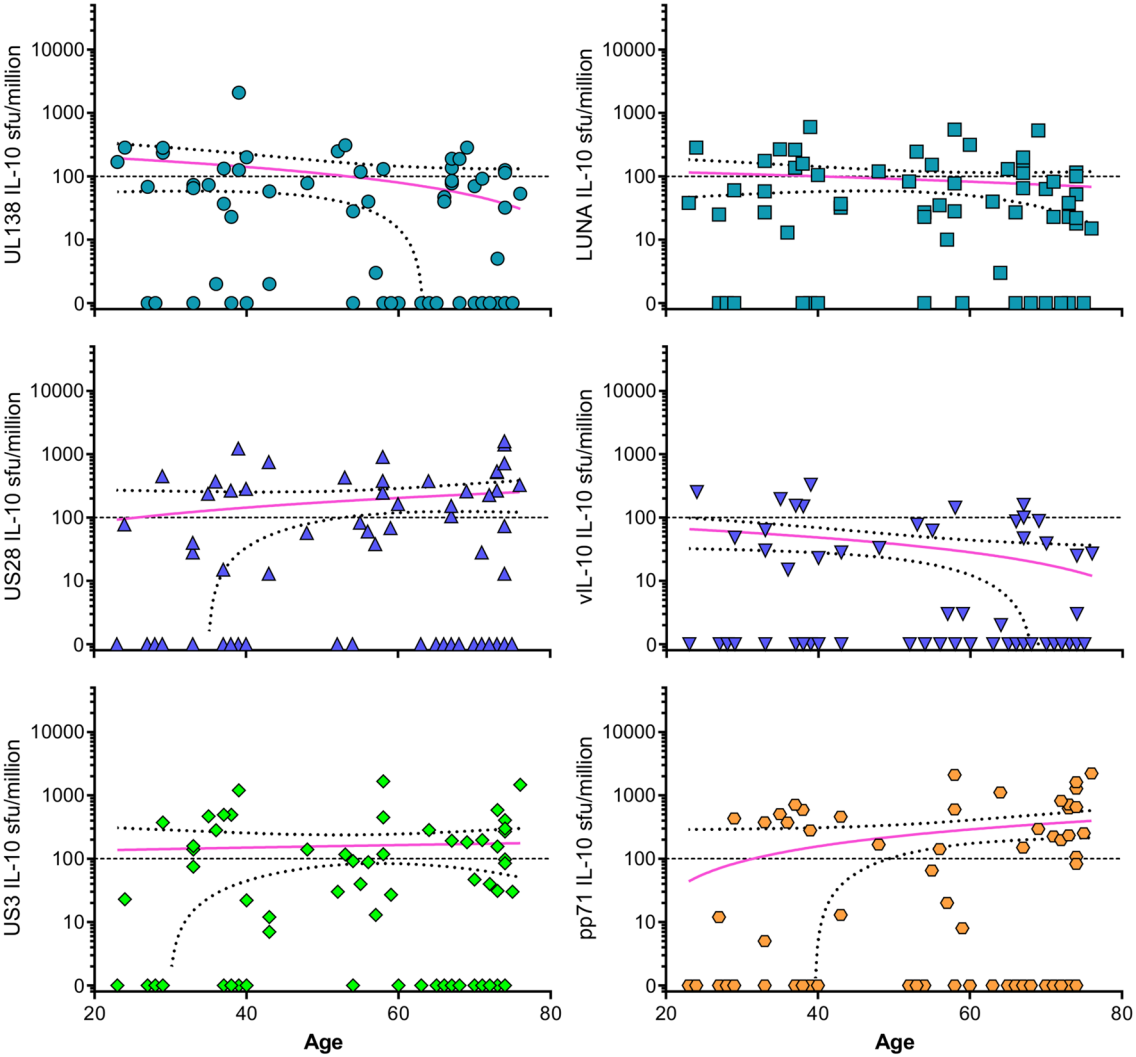
CD4 + T cell IL-10 responses to UL138, LUNA, US28, vIL-10, US3 and pp71 HCMV proteins. The CD4 + T cell IL-10 responses measured by FluoroSpot versus donor age from the ARIA study [23] to six HCMV proteins UL138 (turquoise circles), LUNA (turquoise squares), US28 (blue triangles), vIL-10 (blue inverted triangles), US3 (green diamonds) and pp71 (orange hexagons) are shown. There is no significant correlation (Spearman rank correlation test) between the magnitude of the IL-10 response with donor age for any of the six proteins shown

Following our previous discovery of CMV-specific CD4 + T cells secreting IL-10 in response to two proteins associated with latent infection, UL138 and LUNA [22], and the evidence that there are suppressive and T regulatory cells present in CMV infection, we hypothesised that lifelong carriage of HCMV infection could create an environment where IL-10 secretion was increased by skewing the CD4 + T cell viral response towards a more suppressive phenotype. Furthermore, modulation of the immune response to cytomegalovirus in old age could explain the observation of CMV-DNA present in urine and blood of older donors [92, 101]. We measured CD4 + T cell IL-10 responses to a range of HCMV proteins in a large donor cohort [23] as described in the previous section. When looking at the responses to the individual proteins which generated the most frequent IL-10 responses amongst the donor cohort, UL138, LUNA, US28, vIL-10, US3 and pp71 (Fig. 3), we do not observe either inflation or deflation of these responses, similar to those observed to the cumulative results presented in [23].

An important consideration when looking for parallels between MCMV and HCMV studies is the compartment where responses are being studied. Human studies are mainly limited to looking at immune responses present in the peripheral blood compartment at one moment in time; however, most of the memory inflation populations have been identified in multiple tissue sites of the mouse including the lung [25, 33, 34, 37], spleen [28, 31] and lymph nodes amongst many sites [26, 72, 94]. Murine work on IL-10 responses has also demonstrated that the salivary gland is an important site for observing these CMV-specific CD4 + T cells [96, 97]. It may be that the studies in humans trying to identify IL-10 CMV-specific responses in peripheral blood have not necessarily been using the correct compartment, although clearly any cross-sectional investigation of HCMV-specific T cell frequencies in tissue sites is technically difficult let alone longitudinal studies which are probably impossible.

## Are there tissue-resident HCMV-specific T cell responses?

Interrogating tissue-resident immune responses in humans is clearly technically challenging, with most studies focus being on the peripheral blood compartment because it is easy to access and not detrimental to the health of the donor. Other tissue sites can be accessed during medical procedures and investigations; however, this may mean that the donor is unwell, although depending on individual symptoms the samples obtained may still be useable. CMV and CMV-specific T cell responses have been investigated in samples acquired via other medical procedures, including intestinal IL-10 CD4 + T cells specific for HCMV [96], alveolar macrophages from bronchoalveolar lavage have been shown to be sites of HCMV reactivation in vivo in the lung [102]. Secondary lymphoid organs including, lymph nodes, spleen, bone marrow and tonsils are often removed for a variety of medical reasons, and these tissue sites have also been examined for anti-viral T cell responses [103–106]. Cadavers have been used to access multiple tissue sites from the same donor and recently CD8 + T cell responses, specific for pp65 and IE proteins, have been examined in 24 CMV sero-positive donors at multiple tissue sites including blood, bone marrow, spleen, multiple lymph nodes and mucosal sites including the lungs and intestines. CMV-specific CD8 + T cells were identified at high frequency at several tissue sites including the lungs, blood, bone marrow and lymph nodes [107]. CD8 + T cell responses specific to pp65 and IE1 have also been observed in paired blood and lymph node samples from living donors, examination of clonal responses has shown that during CMV reactivation new clones generated in the peripheral blood do not arise from the lymph node [104], the CD8 + CMV-specific T cells present in the lymph node are generally polyfunctional memory cells, with only low expression of CX3CR1 [103]. CMV-specific CD8 + T cell responses have also been investigated in bone marrow specimens, this study showed an increase in effector memory CD8 + T cells in bone marrow samples, although the frequency of CMV-specific T cells was lower in the bone marrow compared to peripheral blood in this donor cohort [106].

**Fig. 4:**
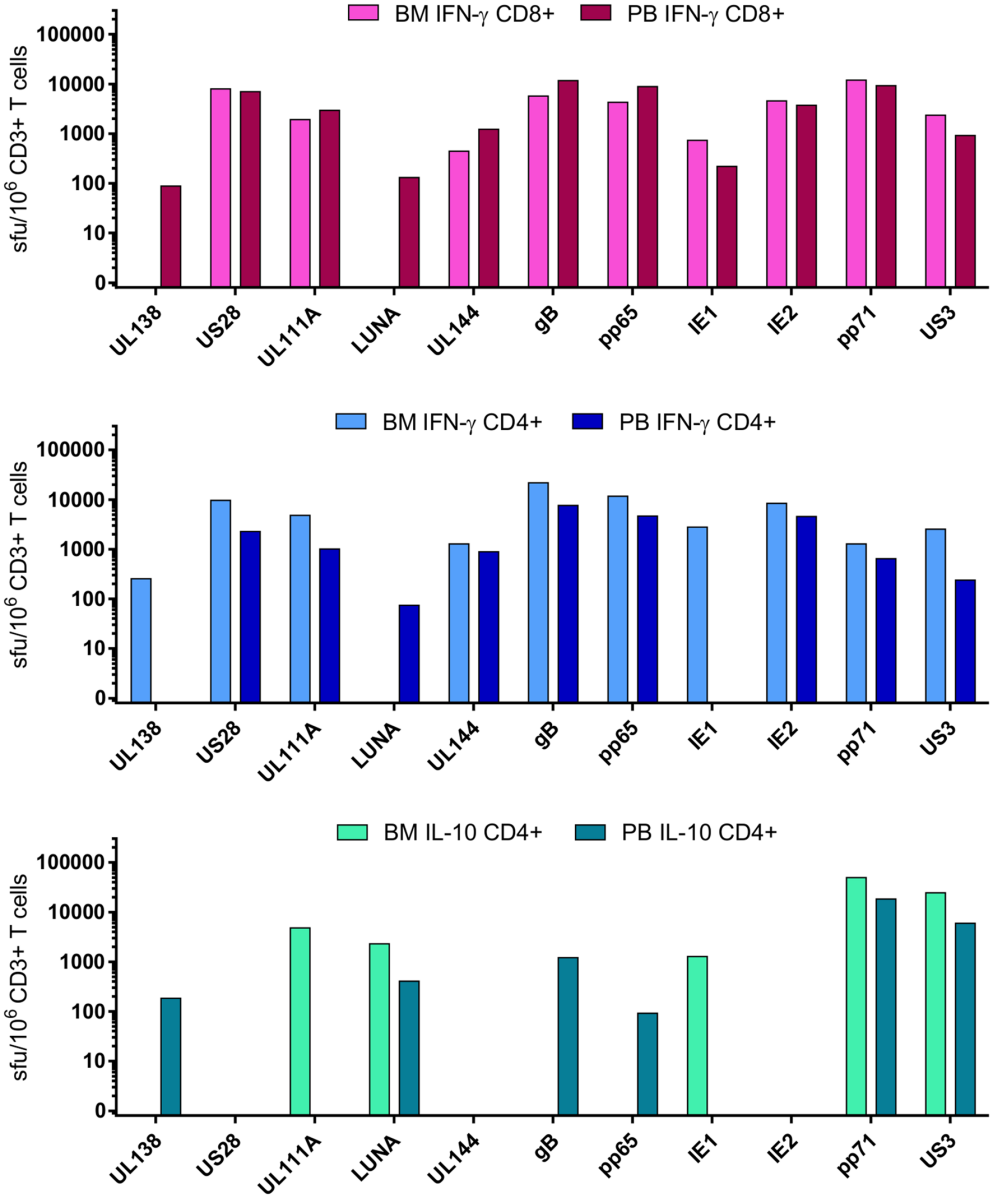
CD8 + T cell IFN-γ responses and CD4 + T cell IFN-γ and IL-10 responses in paired peripheral blood and bone marrow samples. IFN-γ and IL-10 T cell responses to 11 HCMV proteins were measured by FluoroSpot as previously described [23] in peripheral blood and bone marrow sample from the same donor. The CD8 + T cell IFN-γ (top graph—pink bars), the CD4 + T cell IFN-γ (middle graph—blue bars) and the CD4 + T cell IL-10 (bottom graph—turquoise bars) responses in the two compartments are shown

Most studies of HCMV-specific T cells so far in tissue sites have neglected CD4 + T cell responses and functional responses, relying on phenotyping the total population and specificity by tetramers containing pp65 and IE1-derived peptides to quantify the antigen-specific cells. However, it is clear that there are HCMV CD4 + and CD8 + T cell responses to a much broader range of proteins than these two immunodominant proteins [22–24, 50, 68–70]. We have conducted preliminary studies looking at CD4 + and CD8 + T cell responses isolated from paired peripheral blood and bone marrow samples to 11 different HCMV proteins measuring IFNγ and IL-10 responses by FluoroSpot (Fig. 4). The results from the CD8 + T cell compartment show that IFNγ responses to all 11 proteins are detectable in the peripheral blood, but there are no detectable CD8 + T cell responses to UL138 and LUNA in the bone marrow specimen. When looking at CD4 + T cell responses both IFNγ and IL-10 responses show a distinct profile in the bone marrow cells compared to peripheral blood, for instance, there is an IL-10 response to IE1 in the bone marrow sample, we have rarely observed IL-10 responses to this protein in peripheral blood [23]. Also, the IFNγ CD4 + T cell response to the latency-associated proteins is at a higher frequency in the bone marrow sample compared to the peripheral blood sample. This initial result suggests that it will be important to look for both IFNγ and IL-10 T cell responses to HCMV in sites other than peripheral blood.

## The importance of understanding anti-viral functional responses in HCMV

The studies into HCMV immune responses discussed previously have relied on identification of responses by tetramer flow cytometry, peptide or viral lysate stimulation in ELISPOT or intracellular cytokine assays, as such this studies antigen stimulation in the absence of intact functional virus expressing immune evasion molecules [5]. This contrasts with the numerous murine studies, where the “memory inflation” phenomenon can be interrogated using viral strains with inflationary or immune evasion proteins mutated and in mice which have also been genetically manipulated to remove specific immune cell populations or irradiated and specific T cell populations adoptively transferred. In humans, the immune responses we observe are a result of infection with a “wild-type” virus and the effects of viral immune evasion, manipulation and modulation of the anti-cytomegalovirus immune response most likely enables the virus to persist and establish latent infections [4]. However, we are not able to manipulate either the viral encoded factors or the host immune response in vivo as is possible in murine models. To improve our understanding of the generation, maintenance and functionality of HCMV-specific T cell responses require development of appropriate in vitro experimental models, where gene editing approaches and analysis of isolated immune cell populations can be performed.

**Fig. 5:**
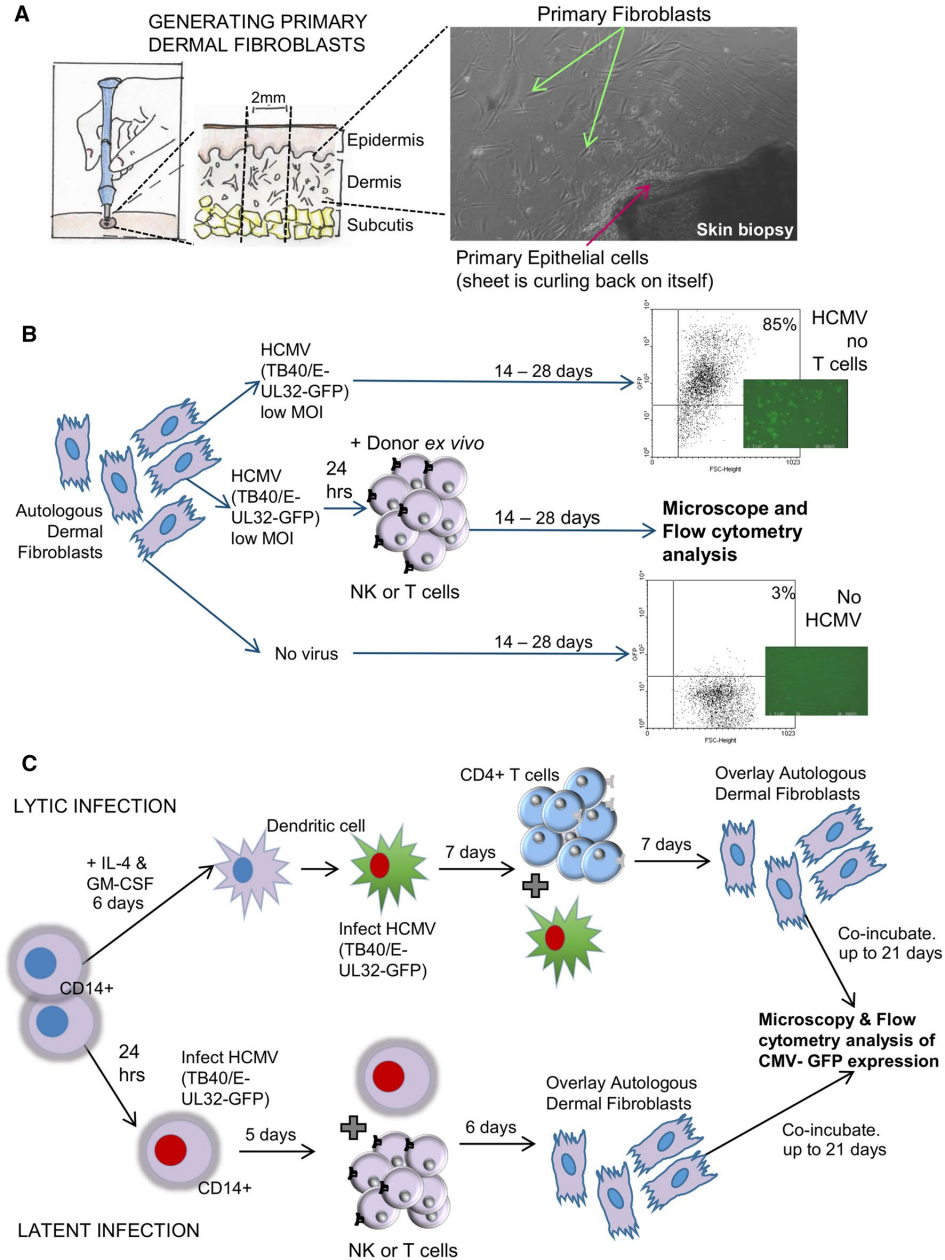
Illustration of the viral dissemination assay model system. A schematic of the generation of autologous primary dermal fibroblasts from a 2-mm punch skin biopsy sample and a microscope image (×10 magnification—bright field) of the fibroblasts growing from a portion of the skin biopsy are shown (**a**). A pictorial representation of the experimental protocols of the viral dissemination assay (**b**) and the myeloid cell-based modified viral dissemination assay (**c**) are shown

We have developed a viral dissemination assay utilising primary dermal fibroblasts from individual donors, grown from a 2-mm punch biopsy (Fig. 5a), which allows the measurement of CD8 + T cell anti-viral functionality in a fully autologous system (Fig. 5b) [68], and we have modified the assay to allow the interrogation of NK cells [108] and both CD4 + and CD8 + T cells anti-viral responses against lytic infection in myeloid cells [24]; furthermore, the myeloid cell-based assay will also allow the interrogation of immune cell interactions with experimental latent infection in monocytes (Fig. 5c).

The viral dissemination assay has shown that pp65, IE1, pp71 and US3 specific CD8 + T cells are able to control the spread of the virus, this direct anti-viral control is present in CMV-specific CD8 + T cells isolated directly ex vivo from peripheral blood [68]. Using a version of this assay (Fig. 5b), the role of NK cells expressing the inhibitory receptor LIR1 to control viral spread was elucidated [108]. Ongoing investigations have shown that CMV-specific CD8 + T cell responses in isolation are effective in preventing the spread of the virus encoding a full complement of immune evasion molecules, by keeping the virus static in cells, but removal of CD8 + T cells from the dissemination assay allows the virus to restart spreading through the dermal fibroblast monolayer. However, when using a mutant cytomegalovirus with US2-11 genes deleted in the viral dissemination assay, this HCMV gene region encodes immune evasion molecules that interfere with antigen processing and presentation pathways [5], ex vivo CD8 + T cells alone are virucidal (data not shown). Co-culture of whole PBMC with clinical strains of the virus (expressing all immune evasion genes) was virucidal. We hypothesise that the interaction between the different immune cell subsets (CD4/8 + T cell, NK cells and monocytes) augments the cell-mediated response overcoming virus-mediated immune evasion and leading to killing of virus-infected cells. The mechanism of this virucidal activity is currently being investigated.

Using the modified viral dissemination assay (Fig. 5c), we have shown that CD4 + T cells isolated from CMV sero-positive donors are able to control viral dissemination at very low effector to target (E:T) ratios and that CD4 + T cells from CMV sero-negative donors cannot [24]. In this study we had one donor aged over 70 years, and at the lowest E:T ratios their CD4 + T cells were not controlling viral dissemination as efficiently as the four younger donors analysed, suggesting that there may be a slight loss of quality in the functional control of viral dissemination in older CMV sero-positive donors. The loss of quality of the anti-cytomegalovirus immune response in older donors may explain why reactivating virus is detected in urine from older and not younger donors [92]. Utilising both the fibroblast and myeloid cell-based viral dissemination experimental models will enable the interrogation of whether tissue resident anti-HCMV T cells are more effective at controlling viral dissemination than T cells isolated from peripheral blood, and whether different T cell memory populations (e.g., effector memory or CX3CR1 + memory) have differing abilities to control the spread of or kill HCMV-infected cells.

## Conclusion

Investigation of T cell responses to HCMV are challenging but appropriate study design such as conducting more longitudinal studies in a wider range of donor cohorts will help to elucidate whether “memory inflation” is a phenomenon that occurs. Also these studies will enable a greater understanding of how HCMV-specific T cell memory evolves over time in the human host. Furthermore, increasing the breadth of cytomegalovirus protein responses analysed including using intact wild-type virus in assays that measure anti-viral effector function will more closely replicate the work performed in the MCMV model system. Performing analyses of T cell responses from a wider range of tissue sites and utilising a range of functional outputs in the face of viral immune evasion strategies will increase our understanding of the maintenance of the immune response to HCMV in both lytic and latent infection. Such a fundamental understanding of the immunobiology of HCMV should inform strategies to better target the virus and the possibility of removing latent virus-infected cells to reduce complications of virus reactivation in the immunosuppressed transplant setting.
